# Protein and Amino Acid Supplementation Does Not Alter Proteolytic Gene Expression following Immobilization

**DOI:** 10.1155/2011/539690

**Published:** 2011-08-09

**Authors:** Jennifer A. Bunn, Thomas W. Buford, Monica C. Serra, Richard B. Kreider, Darryn S. Willoughby

**Affiliations:** ^1^Exercise and Biochemical Nutritional Laboratory, Department of Health, Human Performance, and Recreation, Baylor University, Waco, TX 76798, USA; ^2^University of Florida College of Medicine, Gainesville, FL 32611, USA; ^3^Education and Clinical Center of the Baltimore Veterans Affairs Medical Center, University of Maryland School of Medicine and the Geriatric Research, Baltimore, MD 21201, USA; ^4^Texas A & M University, College Station, TX 77843, USA

## Abstract

*Objective*. To determine if supplementation of protein and amino acids (PAA) decreases skeletal muscle expression of atrophy-related genes, muscle mass, and strength during immobilization in humans. *Methods*. Twenty males wore a lower-limb immobilization boot for 28 days and consumed either a PAA supplement (28 g protein) or carbohydrate placebo (28 g maltodextrose), while consuming their normal daily diet. Testing sessions included dietary analysis, lower-leg girth and body composition measurements, strength testing, and gastrocnemius muscle biopsies. Muscle was analyzed for mRNA expression of markers in the ubiquitin and calpain systems, myostatin, TNF-**α**, and NF-**κ**B. *Results*. All genes of interest increased over time (*P* < .05), but there was no difference between groups. Lower-leg girth decreased over time (*P* = 0.02); however, there were no significant changes in body composition or strength. *Conclusion*. Short-term lower-limb disuse, despite the absence of significant muscle atrophy, is associated with increases in skeletal muscle gene expression of several proteolysis-related genes. These changes do not appear to be altered by oral PAA supplementation.

## 1. Introduction

Muscle atrophy is characterized by a reduction in muscle protein synthesis or an increase in protein degradation [1–4] that results in decreased fiber diameter, force production, and fatigue resistance [5]. Muscle atrophy is often caused by injury [6], fasting [7–11], disease [12–21], or immobilization [1, 8, 10, 11, 22–27]. In all forms of muscle atrophy, there appears to be a shift in the balance between synthesis and degradation, but during unloading and disuse conditions, the decrease in protein synthesis appears to drive the loss of muscle mass, while the rate of protein degradation remains fairly constant [28, 29]. 

During limb immobilization, the rate of protein synthesis and degradation is affected by the removal of a mechanical stimulus [30], thereby triggering the ubiquitin proteolytic system (UPS), calcium-activated protease system (CAS), and myostatin to carry out skeletal muscle proteolysis. Protein degradation via the UPS is carried out by the ubiquitin molecule and a series of enzymes (E1, E2, E3) tagging a specific protein for destruction. The protein is then taken into the 26S proteasome where degradation occurs [31, 32]. Calpains mediate skeletal muscle degradation through digestion of individual myofibrillar proteins [33–37] and tend to influence gene expression through the cleavage of specific transcription factors, thereby affecting the UPS [38]. Research regarding myostatin suggests that expression may be associated with muscle myosin heavy chain isoform IIb [25], as Willoughby et al. [22] noted a 125% increased expression of myostatin mRNA in the immobilized human gastrocnemius, which is composed of approximately 76% of type II muscle fibers [39].

Currently, there is no evidence supporting the involvement of tumor necrosis factor-*α* (TNF-*α*) in disuse atrophy [5]. However, administration of TNF-*α* has been shown to activate cachexia-induced protein degradation [40, 41] and increase ubiquitin mRNA expression and activity of ubiquitin conjugates in skeletal muscle. TNF-*α* also activates nuclear factor-*κ*B (NF-*κ*B), which is involved in immune and inflammatory responses [42], and has been shown to increase myofibrillar proteolysis and suppress myosin synthesis via the UPS [12, 40, 43–45]. Together, TNF-*α* and NF-*κ*B upregulate E2 and the 20S proteasome in the UPS during diseased states [5, 41, 46, 47]; however, current research shows that TNF-*α* is unchanged with disuse-induced atrophy in humans even though NF-*κ*B levels are markedly increased [12, 42, 48]. This suggests that disuse-induced atrophy may activate NF-*κ*B through an alternative pathway in humans. 

Protein and amino acid (PAA) supplementation has often been used as a nutritional countermeasure to augment muscle protein synthesis when taken immediately before and after resistance exercise [49–55], as well as attenuate muscle protein degradation [56–58]. However, literature supporting protein supplementation during disuse atrophy is lacking and the extent to which protein quality and amino acid profile affect efficacy of protein supplementation is unknown. Willoughby et al. [53] assessed the effects of resistance training and a PAA supplement, identical to the one used in this study, and results indicated that PAA supplementation induced greater increases in bench press, leg press, myofibrillar protein, and type IIa myosin heavy chain expression. In fact, chronic ingestion of protein and/or essential amino acids may help attenuate muscle atrophy and muscle protein breakdown during disuse [3, 4, 59, 60], as well as exhibit greater weight gain over nonsupplemented patients during disease [61], and preserve muscle strength after bed rest [62]. The purpose of this study was to investigate the effects of PAA supplementation on muscle degradation and expression of muscle proteolytic-related genes resulting from 28 days of gastrocnemius muscle disuse by way of lower-leg/ankle immobilization using a lower-leg walking boot.

## 2. Methods

### 2.1. Participants

Twenty apparently healthy, recreationally active males (18–30 years of age) participated in the study. Only participants considered as either low or moderate risk for cardiovascular disease, with no contraindications to exercise, and who had not consumed any nutritional supplements one month prior to the study were enrolled. All eligible participants provided informed written consent based on university-approved documents. Participants were instructed to refrain from exercise for 48 hours, fast for eight hours, and record dietary intake for four days prior to each testing session at days 0, 14, and 28. Participants' diets were not standardized, and they were asked not to change their dietary habits during the course of the study. The four-day dietary records were evaluated with the ESHA Food Processor 8.6 (ESHA Research, Salem, Ore, USA) dietary assessment software program to determine the average daily macronutrient consumption.

### 2.2. Supplementation

Participants were matched according to total body mass and assigned into either the placebo (PLA) or PAA supplement group in a double-blind fashion. Supplements were independently prepared and appropriately blinded in identical containers by FSI Nutrition (Omaha, Neb, USA) and were identical in appearance, smell, and taste. Participants consumed a total of three daily doses of PAA or PLA in 500 mL of water. Per dose, the PAA supplement included 28 g protein (14 g whey protein, 6 g whey protein isolate, and 4 g milk protein isolate, 4 g calcium caseinate) and 12 g free amino acids (0.22 g arginine, 0.22 g histidine, 0.14 g isoleucine, 6.0 g leucine, 0.44 g lysine, 0.44 g methionine, 0.20 g phenylalanine, 0.22 g valine, 0.12 g aspartate, 2.0 g glutamine, and 2.0 g tyrosine). Per serving, the PLA included 28 g of maltodextrose.

### 2.3. Lower-Leg Immobilization Protocol

One leg of each participant was randomly chosen for immobilization. The gastrocnemius was immobilized in a neutral position using a lower-limb walking boot (Ultra-4 ROM Walker, DJO, Vista, Calif, USA) by stabilizing the talocrural joint at zero degrees of plantar flexion [22], verified through goniometric measurement. The immobilization boot was worn for 28 days, and participants were allowed to remove the boot only for bathing, sleeping, and during visits to the laboratory at days 14 and 28 for the testing sessions. Participants documented the amount of time they spent out of the boot.

### 2.4. Body Composition, Girth, and Strength Measurements

Body composition, girth, skinfold, and strength measurements were determined at days 0, 14, and 28. Dual energy X-ray absorptiometry (DEXA) (Hologic, Waltham, Mass, USA) was used to determine body composition. Within the whole body scan, a subsection including the knee joint line to the lateral/medial malleolus was isolated for the determination of lower leg muscle mass consisting of the gastrocnemius. Lower-leg girth was assessed by measuring the diameter at the point of the largest protrusion of the gastrocnemius. The distance between the largest protrusion of the gastrocnemius and the midpoint of the medial malleolus was measured at baseline to ensure that the gastrocnemius girth was measured at the same point at subsequent visits. Lower-leg skinfold measurements were performed using standard procedures at the same site on the leg, using Lange skinfold calipers (Beta Technology, Santa Cruz, Calif, USA). All girth and skinfold measurements were performed in triplicate. 

The Biodex Isokinetic Dynamometer (Medical Systems, Inc., Shirley, NY, USA) was used to assess plantarflexion maximal voluntary contraction (MVC) isometrically at 30 degrees of plantar flexion. One five-second isometric contraction was used for a warm-up, followed by a one-minute rest period. Participants performed three maximum effort repetitions held for five seconds each, separated by one minute of rest. The peak value of the three repetitions was recorded as the MVC value. 

### 2.5. Muscle Biopsies and Analysis

Percutaneous muscle biopsies (approximately 10–20 mg) were obtained from the medial head of the gastrocnemius of each participants' leg at a depth between 0.5 and 1 cm using the True-Core II (Angiotech Pharmaceuticals, Vancouver, British Columbia, Canada). Samples were extracted under local anesthesia from the middle portion of the belly of the medial gastrocnemius muscle. The tissue sample was flash frozen in liquid nitrogen and stored at –80°C for future analyses. For subsequent biopsies, attempts were made to extract tissue from the same location by using the prebiopsy scar, depth markings on the needle, and a successive puncture that was made approximately 0.5 cm to the former from medial to lateral. Muscle samples were obtained at days 0, 14, and 28.

Approximately 10 mg of muscle tissue was used for analysis. Based on previous guidelines [53, 63], each muscle sample underwent total RNA isolation, reverse transcription, cDNA synthesis, and real-time PCR amplification and quantitation. RNA and cDNA concentrations were determined spectrophotometrically (Helio *γ*, Thermo Electron, Milford, Mass, USA) by optical density (OD) at 260 nm using an OD_260_ equivalent to 50 *μ*g/*μ*L and 40 *μ*g/*μ*L [64], respectively, and the final concentration was expressed relative to muscle wet weight. The starting cDNA template concentration was standardized by adjusting all samples to 200 ng prior to amplification [53].

The mRNA sequences of human skeletal muscle published in the NCBI Entrez Nucleotide database (http://www.ncbi.nlm.nih.gov/) were used to construct oligonucleotide PCR primers using Beacon Designer software (Bio-Rad, Hercules, Calif, USA). The sense and antisense primers were synthesized commercially (Integrated DNA Technologies, Coralville, Iowa, USA). Muscle was analyzed for mRNA expression of ubiquitin, 20S-HC2, 20S-HC3, E2 (class I UbcH5c), E3 (hect-domain UBE3B), atrogin-1, MuRF1, calpain 1, calpain 2, myostatin, TNF-*α*, and NF-*κ*B. *β*-actin was used as an external control standard for each reaction due to its consideration as a constitutively expressed “housekeeping gene,” and the fact that it has been shown to be an appropriate external reference standard in real-time PCR in human skeletal muscle following acute exercise [53]. 

Based on previous guidelines [63], 200 ng of cDNA were added to each of the 25 *μ*L polymerase chain reaction (PCR) reactions using iQ SYBR Green Supermix (Bio-Rad, Hercules, Calif, USA). The specificity of the PCR was demonstrated with absolute negative controls using separate PCR reactions with no cDNA template or primers, and a single gene product was confirmed using DNA melt curve analysis. Aliquots (20 *μ*L) of the PCR mixtures were electrophoresed in 1.5% agarose gels in 1X Tris-Acetate-EDTA (TAE) buffer to verify positive amplification. The gel was stained with ethidium bromide (present in the TAE buffer at 1 *μ*g/mL) and illuminated with UV transillumination (Chemi-Doc XRS, Bio-Rad, Hercules, Calif, USA). Basal gene expression was calculated using the 2^−ΔCT^ method and compared between groups using the nonparametric Mann Whitney test [65]. Fold changes in expression were calculated for each gene of interest (GOI) at days 14 and 28 by the 2^−ΔΔCT^ method [66].

### 2.6. Statistical Analyses

Fold changes were compared between groups for each GOI at days 14 and 28 using the Mann Whitney test [65]. A two-tailed *P* value of <0.05 was used to indicate significance throughout. Relative to hemodynamic, body composition, girth, and strength variables, a 2 (supplement) × 3 (time points) repeated measures multivariate analysis of variance (MANOVA) mixed methods with the repeated measures on the second factor was utilized. For each MANOVA, separate ANOVAs on each dependent variable were conducted as follow-up tests to the MANOVA. In addition, all statistical analyses not meeting the sphericity assumption for the within-subjects analyses, a Huynh-Feldt correction factor was applied to the degrees of freedom in order to increase the critical *F*-value to a level that would prevent the likelihood of committing a type I error. All statistical procedures were performed using SPSS 16.0 software (Chicago, Ill, USA) and a probability level of <0.05 was adopted throughout.

## 3. Results

### 3.1. Participants

Results showed no significant differences between groups at baseline for age, height, total body weight, body composition, diet, or any hematologic variables, indicating homogeneity between groups (Table 1). Table 2 shows the results of the dietary analyses. Baseline dietary analysis showed no significant differences between groups for any variables. The ingestion of PAA and PLA did result in a significant difference between groups for total daily protein intake and relative protein intake (*P* < 0.001). There was also a significant difference for total protein intake and relative protein intake from baseline at days 14 and 28 in the PAA group (*P* < 0.001). There was also no difference between groups or over time for the amount of time spent not wearing the boot (Table 3). 

### 3.2. Body Composition and Strength

There was no difference in body composition and strength measurements between groups or over time, but analyses indicated a significant time effect for leg girth (*P* = 0.02). Further comparisons showed significantly larger girth measurements at baseline than day 14 (*P* = 0.05) and day 28 (*P* = 0.04). Table 4 shows the mean and standard deviation for body composition, girth, and strength variables.

### 3.3. mRNA Expression for Proteolytic Genes

At baseline, no significant difference between groups was observed for any GOI. The effects of immobilization on gene expression were notable as all GOI increased a minimum of threefold (*P* < 0.05) in both groups (Table 5). Supplemental PAA ingestion did not attenuate these increases as no significant difference existed between groups for any GOI at either time point. Most notable of the changes were those of TNF-*α*, E3, myostatin, calpain 2, and ubiquitin. This is the first study to show an increase in the expression of TNF-*α* during disuse-induced atrophy in humans. Increased expression of E3 is noteworthy because E3 is the recognizable factor within the UPS, triggering the 26S proteasome to carry out protein degradation. The increased expression of myostatin further supports the potential association of myostatin to breakdown type II muscle fibers.

## 4. Discussion

The results of this study showed that markers of muscle proteolysis were increased during a period of immobilization, but the ingestion of 84 g/day of additional protein did not attenuate any of the proteolytic effects when compared to placebo. Additionally, the initiation of the proteolytic program was observed without measurable atrophy. The conclusions of this study are therefore based on the genetic changes observed without atrophy. 

In conjunction with previous research, results also indicated that 28 days of immobilization resulted in significant increases in the mRNA expression for the ubiquitin protein, E2, E3, 20S-HC2, 20S-HC3, atrogin-1, and MuRF1 [1, 8, 22, 67, 68]. No other study has shown an increase in the mRNA expression of E3, but this change may indicate a greater need for polyubiquitination of muscle proteins and thus allow for greater protein degeneration. Overall, the results indicate that there appears to be a comprehensive increase throughout the entire UPS pathway. Additionally, the increased expression of calpain 1 and calpain 2 agrees with previous studies indicating that calpains affect muscle protein degradation [33, 35, 36]. This finding indicates that calpains play a role not only in protein degradation in diseased states [69] and during damaging exercise performance [70], but also during disuse atrophy. 

No previous study has shown evidence that TNF-*α* is involved in disuse atrophy in humans [5]; instead, it has been shown to activate NF-*κ*B in several diseased states resulting in muscle atrophy [14, 41, 71, 72]. Researchers speculated that NF-*κ*B worked independently from TNF-*α* during immobilization in humans because evidence showed no change in TNF-*α* during unloading, yet still resulted in an increase in NF-*κ*B [12, 42, 48] and atrophy. However, data from the current study indicate that both TNF-*α* and NF-*κ*B mRNA levels were increased. It should be noted that with these data an alternative pathway may not be necessary to activate the degenerative effects of NF-*κ*B, but there may also have been an increase in transcription of these markers without an increase in translation, or perhaps an increase in these markers, without activation. Therefore, more human research is necessary in order to observe this potential association between the UPS and TNF-*α* and NF-*κ*B in order to further elucidate the route by which the cytokines function as signaling mediators of muscle protein degradation. 

In agreement with Reardon et al. [26], increases in mRNA expression of myostatin seen in the present study indicate a strong influence of myostatin on the inhibition of muscle growth as a result of immobilization. However, these results are conflicting with Jones et al. [1] and McMahon et al. [73] who did not observe any changes in myostatin during hind-limb unloading. Myostatin may be a fiber type-specific inhibitor of muscle growth, suggesting a stronger association between myostatin and type IIb muscle fibers [25]. The gastrocnemius is composed of about 50% slow twitch fibers and 50% fast twitch fibers [74], indicating a greater amount of fast twitch fibers than that of the soleus and digitorum longus studied by McMahon et al. [73]. However, Jones et al. [1] showed no changes in myostatin in the vastus lateralis, which is composed of approximately 68% type II fibers [74]. Overall, the conflicting evidence of myostatin's role within muscle atrophy during immobilization renders the need for more research in order to show specific effects and action on the various muscle fiber types. 

The results of this study showed that the PAA supplement ingested was not influential in attenuating the expression of myostatin and the ubiquitin and calpain proteolytic pathways. PAA supplementation has been effective in maintaining a higher protein fractional synthesis rate during disease-induced and unloading-induced proteolysis [4, 59, 60, 75] and following damaging eccentric exercise by stimulating muscle protein synthesis, but showed little to no effect on muscle protein degradation [49, 51]. Results from the current study could be different from existing literature because ingestion of extra dietary PAA may not effectively alter the physiological changes in the body that are induced by muscle unloading. It is suggested that amino acid catabolism and whole body protein turnover is upregulated during unloading and immobilization [2], and in as little as 14 days of bed rest whole body protein turnover decreases by 15%, with 50% of the decrease coming from an attenuation of protein synthesis [29]. Research also indicates that during unloading and disuse conditions, the decrease in protein synthesis appears to drive the loss of muscle mass, while the rate of protein degradation remains fairly constant [28, 29]. While there appeared to be no effect on net protein turnover as observed through maintenance of leg lean mass, this may indicate a potential benefit of PAA or PLA on protein synthesis. Previous research [76] has shown that ingesting 1.5 g of protein per kg of body weight per day was the optimal protein intake in the prevention of sarcopenia in the elderly. The participants in the PAA group exceeded this amount with their dietary intake and ingestion of the PAA supplement (Day 14 mean intake 2.2 ± 0.7 g/kg/d; Day 28 mean intake 2.2 ± 0.6 g/kg/d). This information suggests that the optimal protein intake for younger males aged 18–30 years old may need more daily protein than the elderly to prevent muscle loss during immobilization. Thus, the amount of dietary protein ingested may not have been enough to attenuate protein synthesis and effectively alter proteolytic pathways during immobilization.

Lower-limb girth decreased in the immobilized limb from baseline at days 14 and 28, but the results from the DEXA did not indicate a significant change from baseline in lean mass in the lower leg after immobilization. Muscle mass should have decreased throughout the 28 days [1], but these results may be due to the margin of error that is displayed by the DEXA. The accuracy of the DEXA is generally ±2% for fat mass, lean mass, and total mass as assessed by direct comparison with hydrodensitometry and scale weight, but these values are for whole body assessment, not assessment of the subcompartments. Results also showed no significant changes in isometric muscular strength in the lower leg as measured by peak torque and average torque. The DEXA and strength results may differ from previous studies because the present study employed functional lower-limb immobilization through a walking boot, rather than complete bed rest or limb suspension. The current body of information regarding atrophy is largely derived from animal models and bed rest studies, so the results observed in the present study may differ from previous literature because the limb was placed in a functional walking boot rather than completely immobilized via bed rest or hind-limb suspension. This *modified* immobilization may have influenced the unexpected results in leg lean mass. Furthermore, because the participants' contralateral limb remained fully functional, there may have been a cross-transfer effect that took place between the immobilized and free limb [77]. The lack of measurable atrophy and inclusion of plasma amino acid analysis is viewed as a limitation of the study.

## 5. Conclusion

While this study did not present any significant data indicating the potential inhibitory effects of ingesting a PAA supplement when undergoing skeletal muscle unloading, it did support previous research indicating the various proteolytic genes that are upregulated during muscle atrophy. Myostatin, TNF-*α*, NF-*κ*B, and markers within the UPS and CAS were increased with immobilization, but the ingestion of 84 g/day of additional protein did not attenuate any of the proteolytic effects compared to the PLA. TNF-*α* was shown to be upregulated in immobilization, indicating the need for further human studies to evaluate the connection between TNF-*α* and NF-*κ*B during muscle disuse. The results also support previous studies that myostatin is effective in inhibiting muscle growth in association with immobilization. It should be noted that the authors do not believe that these changes are results of repeated muscle biopsies of the same area. It has been documented that gene expression from acute trauma, like a muscle biopsy, peaks four to 12 hours later and declines full 24–48 hours after exercise [78]. Additionally, the muscle biopsies were separate by two weeks, so the repeated bout effect would not apply. 

Future studies should take into consideration other potential therapeutic options that may attenuate these proteolytic pathways, because muscle wasting is often associated with increased morbidity and mortality. While exercise is a viable therapy for most disease-induced muscle wasting disorders, it is not an option for those undergoing muscular immobilization.

## Figures and Tables

**Table 1:** Baseline participant demographic data for placebo (PLA) and protein/amino acid (PAA) groups. All data are presented as mean ± standard deviation. Results indicated no significant differences between groups at baseline or throughout the course of the study (*P* > 0.05).

Variable	PLA	PAA
Age (years)	20.9 ± 2.7	20 ± 1.5
Body weight (kg)	80.68 ± 13.79	82.28 ± 16.35
Total body water (liters)	42.13 ± 5.36	42.63 ± 6.77
Lean mass (kg)	55.63 ± 8.15	55.60 ± 9.67
Fat mass (kg)	15.65 ± 6.77	16.41 ± 6.94
% mody fat	20.6 ± 6.76	21.41 ± 5.74
Heart rate (bpm)	66.6 ± 10.07	69.6 ± 6.85
SBP (mm Hg)	116.3 ± 12.88	117.5 ± 8.26
DBP (mm Hg)	74.7 ± 6.93	77.0 ± 7.73
Avg. daily calories (kcal)	2400 ± 861	1936 ± 236
Avg. daily CHO (g)	289.5 ± 103.4	232.1 ± 33.1
Avg. daily fat (g)	105.3 ± 52.0	80.9 ± 16.9
Avg. daily protein (g)	94.0 ± 42.5	72.6 ± 12.3

**Table 2:** Dietary values assessed prior to the three testing sessions for placebo (PLA) and protein/amino acid (PAA) groups. All data are presented as mean ± standard deviation. Results indicated a significant difference between groups at days 14 and 28 for total protein and relative protein (*P* < 0.001) and a significant change from baseline over time for the PAA group (*P* < 0.001). *Denotes a significant time effect and ^†^denotes a significant difference between groups.

Variable	PLA			PAA		
Day	0	14	28	0	14	28

Total calories (kcal/day)	2400 ± 861	2309 ± 690	2292 ± 659	1936 ± 236	2494 ± 591	2343 ± 589
Total CHO (g/day)	290 ± 103	310 ± 90	308 ± 88	232 ± 33	257 ± 43	241 ± 69
Total fat (g/day)	105 ± 52	76 ± 28	81 ± 31	81 ± 17	89 ± 33	81 ± 34
Total protein (g/day)^†^	94 ± 43	78 ± 22	82 ± 27	73 ± 12	177 ± 36*	167 ± 26*
Relative protein (g/kg/day)^†^	1.2 ± 0.4	1.0 ± 0.3	1.0 ± 0.4	0.9 ± 0.3	2.2 ± 0.7*	2.1 ± 0.7*

**Table 3:** Average time spent out of the ankle immobilization boot for placebo (PLA) and protein/amino acid (PAA) groups. All data are presented as mean ± standard deviation. Results indicated no significant differences throughout the course of the study (*P* > 0.05).

Variable	PLA		PAA	
Days	0–14	14–28	0–14	14–28

Total time (hours)	127.6 ± 14.3	123.2 ± 12.0	132.2 ± 27.2	133.3 ± 19.3
Avg. daily time (hours)	9.1 ± 1.0	8.8 ± 1.5	9.5 ± 2.0	9.5 ± 1.4
Avg sleep time (hours)	8.6 ± 1.0	8.3 ± 0.8	9.0 ± 1.8	9.0 ± 1.2
Avg time awake (hours)	0.5 ± 0.2	0.5 ± 0.2	0.4 ± 0.2	0.5 ± 0.2

**Table 4:** Lower leg girth, skinfold, and body composition measurements for placebo (PLA) and protein/amino acid (PAA) groups. All data are presented as mean ± standard deviation. *Denotes a significant time effect.

Variable	PLA				PAA	
Day	0	14	28	0	14	28

Girth (cm)*	36.0 ± 2.0	35.4 ± 2.0	35.1 ± 2.1	35.4 ± 1.8	35.0 ± 1.7	34.7 ± 1.8
Skinfold (mm)	13.0 ± 4.0	13.0 ± 4.0	13.0 ± 3.0	14.0 ± 4.0	14.0 ± 5.0	14.0 ± 4.0
Leg lean mass (kg)	2.43 ± 0.26	2.44 ± 0.26	2.40 ± 0.28	2.72 ± 0.90	2.44 ± 0.22	2.41 ± 0.22
Leg fat mass (kg)	0.67 ± 0.27	0.64 ± 0.25	0.73 ± 0.38	0.87 ± 0.51	0.72 ± 0.31	0.67 ± 0.33
Peak torque (ft-lbs)	22.6 ± 8.6	19.7 ± 7.7	21.0 ± 9.3	27.6 ± 11.8	26.7 ± 7.9	25.7 ± 10.3
Avg torque (ft-lbs)	19.6 ± 8.4	18.5 ± 7.6	19.2 ± 9.1	25.8 ± 11.1	25.2 ± 7.8	24.0 ± 9.5

**Table 5:** Fold changes from baseline for all genes of interest for placebo (PLA) and protein/amino acid (PAA) groups. All genes of interest showed significant increases over time (*P* < .05), but there was no difference noted between supplement groups. *Denotes a significant time effect.

GENE	PLA		PAA	
Day	14	28	14	28

Calpain1*	9.40 ± 6.67	12.21 ± 7.50	6.00 ± 3.15	10.01 ± 5.26
Calpain2*	4.06 ± 2.34	3.53 ± 1.79	4.71 ± 2.51	11.12 ± 7.10
TNF-α*	4.43 ± 1.82	6.17 ± 2.64	5.86 ± 2.55	4.81 ± 1.45
NF-*κ*B*	5.83± 2.23	9.93 ± 6.67	6.82 ± 3.14	5.98 ± 1.79
Ubiquitin*	6.26 ± 3.51	5.86 ± 3.76	5.64 ± 2.51	13.64 ± 6.67
C2*	8.21 ± 4.59	9.37 ± 5.97	4.73 ± 2.03	10.48 ± 3.92
C3*	9.54 ± 5.66	10.09 ± 5.40	7.32 ± 3.84	8.27 ± 3.53
Atrogin1*	12.13 ± 8.43	12.76 ± 8.95	5.48 ± 3.59	7.04 ± 3.07
MuRF1*	5.19 ± 2.49	3.62 ± 1.98	8.33 ± 3.00	7.84 ± 2.58
E2*	10.76 ± 6.30	10.92 ± 6.46	6.17 ± 3.35	6.67 ± 2.43
E3*	5.82 ± 3.09	10.31 ± 6.33	19.02 ± 9.68	22.92 ± 14.27
Myostatin*	8.98 ± 6.18	16.74 ± 12.25	9.23 ± 5.47	21.85 ± 15.33
